# Analysis of Multifractal and Organization/Order Structure in Suomi-NPP VIIRS Normalized Difference Vegetation Index Series of Wildfire Affected and Unaffected Sites by Using the Multifractal Detrended Fluctuation Analysis and the Fisher–Shannon Analysis

**DOI:** 10.3390/e22040415

**Published:** 2020-04-07

**Authors:** Rui Ba, Weiguo Song, Michele Lovallo, Siuming Lo, Luciano Telesca

**Affiliations:** 1 State Key Laboratory of Fire Science, University of Science and Technology of China, Jinzhai 96, Hefei 230026, China; barui@mail.ustc.edu.cn; 2 Department of Civil and Architectural Engineering, City University of Hong Kong, Tat Chee Avenue, Kowloon, Hong Kong, China; bcsmli@cityu.edu.hk; 3 Agenzia Regionale per la Protezione dell’ Ambiente di Basilicata (ARPAB), 85100 Potenza, Italy; michele.lovallo@alice.it; 4 Consiglio Nazionale delle Ricerche (CNR), Istituto di Metodologie per l’Analisi Ambientale, 85050 Tito (PZ), Italy; luciano.telesca@imaa.cnr.it

**Keywords:** satellite time series, VIIRS, NDVI, wildfires, multifractal detrended fluctuation analysis, Fisher–Shannon analysis

## Abstract

The analysis of vegetation dynamics affected by wildfires contributes to the understanding of ecological changes under disturbances. The use of the Normalized Difference Vegetation Index (NDVI) of satellite time series can effectively contribute to this investigation. In this paper, we employed the methods of multifractal detrended fluctuation analysis (MFDFA) and Fisher–Shannon (FS) analysis to investigate the NDVI series acquired from the Visible Infrared Imaging Radiometer Suite (VIIRS) of the Suomi National Polar-Orbiting Partnership (Suomi-NPP). Four study sites that were covered by two different types of vegetation were analyzed, among them two sites were affected by a wildfire (the Camp Fire, 2018). Our findings reveal that the wildfire increases the heterogeneity of the NDVI time series along with their organization structure. Furthermore, the fire-affected and fire-unaffected pixels are quite well separated through the range of the generalized Hurst exponents and the FS information plane. The analysis could provide deeper insights on the temporal dynamics of vegetation that are induced by wildfire.

## 1. Introduction

The natural phenomenon of vegetation dynamics can reflect the status, types, and changes of terrestrial biomass. The analysis of long-term monitoring data of vegetation helps to better understand the variations in vegetation communities and ecosystems under the abnormal disturbances, especially the wildfires [1]. As one of the most destructive natural hazards, the frequency and severity of wildfires have significantly increased in recent decades with the global warming and climate changing [2,3], which can greatly perturb the vegetation growth and dynamics [4,5]. Therefore, the investigation of the characteristics of vegetation reactions that are provoked by wildfire is imperative for the assessment of vegetation dynamics and resilience [6,7].

Under the influence of wildfires, the vegetation dynamics can be more complicated because of their interactions with biomass, soil, atmosphere, climate, and so on [8]. Deeper investigations need to be conducted at different spatial-temporal scales in conjunction with the local, regional, and global levels in order to assess the impact of fires on the vegetation communities [9]. Fortunately, the development of satellite remote sensing provides an excellent means of the continuous observation for the natural biomass [10,11]. Massive long-term data records are captured via multiple satellite sensors, of which the Suomi National Polar-Orbiting Partnership (Suomi-NPP) satellites were launched on October 28, 2011 as the new generation system to undertake the mission of the previous Moderate Resolution Imaging Spectroradiometer (MODIS) of Earth Observing System (EOS) satellites, which can further provide continuous data records and observations. For the Suomi-NPP sensors, the sensor of Visible Infrared Imaging Radiometer Suite (VIIRS) is designed to collect the measurements of spectral domain from 0.3μm to 14μm for the Earth, which advances its increasing applications in wildfires [12,13].

Various vegetation indices (VI) have been developed to characterize the status of surface biomass, among which the Normalized Difference Vegetation Index (NDVI) [14] is generally used to quantify the status of vegetation. It is defined as the ratio between the reflectance in the spectral domain of visible red and near-infrared; this definition minimizes the noise that is related to different bands and diminishes the effects induced by clouds, shadows, sun, atmospheric attenuation, etc. [15]. The primary land product of VIIRS, the VNP13A1, records the NDVI series since 2012 all over the world, providing abundant data of the long-term satellite time series of the vegetation in the region of interest (ROI).

Various methods were utilized to study the variations and trends of vegetation dynamics induced by wildfires for the purpose of understanding the complexity of ecosystem changing under the external disturbances [16,17]. Lunetta et al. [18] developed an automatic method for annual land cover changes of non-agriculture areas based on MODIS NDVI time series. Besides, fractal analyses, like the Detrended Fluctuation Analysis (DFA), can be used to investigate the characteristics of scaling in non-stationary vegetation time series, providing information on the persistent or antipersistent mechanisms of vegetation dynamics. Telesca et al. [19,20] used the DFA to quantify the persistent behavior of vegetation that is induced by wildfire while using the NDVI time series of SPOT-VGT and MODIS sensors. Li et al. [21] also analyzed the MODIS NDVI series of Daxing’anling region by DFA to assess the fire impact. However only one exponent, namely the DFA scaling exponent, was estimated and a complete characterization of the complex features of vegetation dynamics could not be further explored in these studies. The multifractal analysis, like the Multifractal detrended fluctuation analysis (MFDFA) [22], allows for describing the time dynamics of a series with many exponents, thus providing a more exhaustive description of a complex phenomenon, like vegetation. Recently, Li et al. [21] used a further method to identify in MODIS NDVI time series sites burned by a wildfire in China by using the Fisher–Shannon method, which is based on the joint use of two different statistical methods, Fisher Information Measure (FIM) and the Shannon entropy (SE), and is suited to investigate the characteristics of organization/order of complex time series. The FIM was firstly proposed by Fisher et al. [23] for the analysis of nonstationary signals. Frieden et al. [24] and Martin et al. [25] employed the FIM to analyze the variations of physical systems. The FIM has also been shown to be an efficient tool for the analysis of earthquake-related signals [26,27] and apparent Earth’s resistivity [28]. By the combination of FIM and Shannon entropy power (*N_X_*), being defined through an exponential transformation of SE, the Fisher–Shannon (FS) information plane can be derived, where the discrimination of different dynamics governing the same time series could be carried out. The FS information plane was applied, for instance, to investigate the physical quantities of Langevin equation of friction in earthquake rupture processes [29] and the seismic waves of strong earthquakes [30]. In this paper, the satellite NDVI time series of Suomi-NPP VIIRS that were extracted from four different study sites were analyzed using the MFDFA and FS to investigate the changes of vegetation dynamics that are induced by the Camp Fire (California, USA, 2018). The rest of the paper is structured, as follows: the satellite data, the sites and the statistical methods are described in Section 2. Section 3 and Section 4 present the results of the data analysis and their discussion, respectively, while Section 5 summarizes the conclusions.

## 2. Materials and Methods

### 2.1. Data

The vegetation index NDVI [14] is formulated, as follows:

(1) $$\mathrm{NDVI}=\frac{\rho_{\mathrm{NIR}}-\rho_{\mathrm{red}}}{\rho_{\mathrm{NIR}}+\rho_{\mathrm{red}}},$$

where *ρ*_NIR_ refers to the atmospherically corrected reflectance of the near-infrared band, while *ρ*_red_ represents that of the visible red band.

This index has been the most widely used, because it efficiently measures the status of growth, distribution, and change of the surface biomass [31]. The higher the NDVI value, the better the conditions of vegetation. Since the vegetation can be highly reflectant in the near-infrared spectral domain while being strongly absorbant in the visible red domain, the NDVI value significantly decreases when the vegetation biomass is affected by wildfires as the reflectance of near-infrared band decreases and that of visible red band increases [32]. 

The NDVI time series analyzed in this study are obtained from the Suomi-NPP VIIRS vegetation indices Version 1 data product VNP13A1 [15,33]. The product has been designed to provide global NDVI values since the year 2012 by selecting the optimal available pixel in a frequency of 16 days at the spatial resolution of 500 m. The NDVI were firstly pre-processed to filter out the anomalous pixels that may have been affected by atmosphere interference, cloud contamination, or bidirectional reflectance distribution function (BRDF) effects or a combination of these factors [34]. This pre-processing procedure is based on the combination of the quality information implemented in the VNP13A1 product, which is the pixel reliability metric informing about the quality and usefulness of pixels, the Quality Assessment (QA) metadata objects, and QA science data sets (SDS) that can provide information of VI quality assurance.

In addition, we investigated the departure NDVI (NDVI_d_) series instead of the original NDVI series for the purpose of eliminating the effects of seasonal variations in the NDVI time series. The departure data NDVI_d_ are widely used in the satellite data processing and filtering [19,35,36,37]. The NDVI_d_ is calculated, as follows:

(2) $${\mathrm{NDVI}}_{d}=\mathrm{NDVI}-\mu_{\mathrm{NDVI}},$$

where *μ*_NDVI_ is the average of all samples in all available years that were measured in the same calendar day.

### 2.2. Study Sites

**Figure 1:**
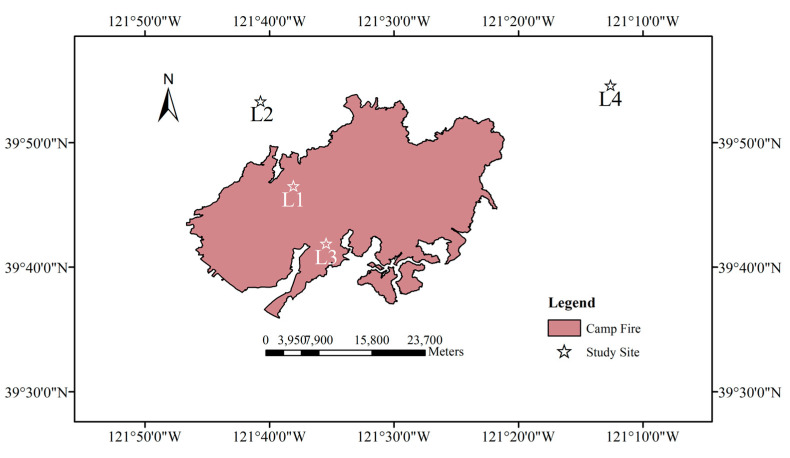
Location of the four study sites (L1, L2, L3, and L4) and the Camp Fire (dark red area).

The Camp Fire occurred in California, USA on November 8, 2018 with the initial coordinates of 39.813° N and 121.435° W. It was the deadliest and most destructive wildfire in California history, being caused by the Pacific Gas and Electric Co. (PG&E) transmission lines and finally killing 85 people [38]. We analyzed the VIIRS NDVI time series from 2012 to 2019 of four study sites to characterize the time dynamics and the fire-affected status of vegetation; two sites were affected by the Camp Fire (L1 and L3), while the other two were not affected (L2 and L4) and located outside the burned area, as shown in Figure 1. For each study site, we extracted 3 × 3 adjacent pixels (P1 to P9). The central coordinates of the four study sites are: L1 (39.776° N and 121.635° W), L2 (39.890° N and 121.630° W), L3 (39.699° N and 121.591° W), and L4 (39.910° N and 121.770° W). Before the occurrence of the wildfire, L1 and L2 were covered by evergreen needleleaf forests, while L3 and L4 by woody savannas (the type of vegetation cover was based on the International Geosphere-Biosphere Programme (IGBP) classification scheme generated from the MODIS Land Cover Product MCD12Q1 [39,40]).

### 2.3. Methods

#### 2.3.1. Multifractal Detrended Fluctuation Analysis

The Multifractal Detrended Fluctuation Analysis (MFDFA) [22] is a well-known method for analyzing the multifractal features of non-stationary signals, like the vegetational series. Given the series *x*(*i*) where *i* = 1, 2, …, *N*, where *N* is its size, *x_ave_* its mean, the profile *y*(*i*) is calculated by integration:

(3) $$y(i)=\sum_{k=1}^{i}\left[x(k)-x_{\mathrm{ave}}\right],$$

The profile *y*(*i*) is then divided into $N_{m}=\mathrm{int}(\frac{N}{m})$ non-overlapping boxes of equal size *m*.

Becasue *N* is usually not a multiple of scale *m*, a small portion of the series at the end would be left. The same data division is performed from the end of the series, thereby finally generating 2*N_m_* segments, in order not to ignore this portion. For each of the 2*N_m_* segments, the polynomial local trend is calculated by a least square fit, and the following variance is obtained:

(4) $$F^{2}(m,\nu )=\left\{ \begin{array}{l} \frac{1}{m}\sum_{i=1}^{m}{\left\{y\left[\left(\nu -1\right)m+i\right]-y_{\nu }\left(i\right)\right\}}^{2},\;\nu =1,\ldots ,N_{m}\\ \frac{1}{m}\sum_{i=1}^{m}{\left\{y\left[N-\left(\nu -N_{m}\right)m+i\right]-y_{\nu }\left(i\right)\right\}}^{2},\;\nu =N_{m}+1,\ldots ,2N_{m} \end{array}\right.,$$

where *y_ν_*(*i*) is the fitting polynomial of *p*-th degree in the segment *ν*. The fitting polynomial of degree *p* eliminates all of the trends in the profile of order up to *p*, thus up to *p* − 1 in the original series [22].

Subsequently, the *q*-th order fluctuation function *F_q_*(*m*) is computed averaging over all the segments, as follows:

(5) $$F_{q}(m)={\left\{\frac{1}{2N_{m}}\sum_{\upsilon =1}^{2N_{m}}{\left[F^{2}\left(m,\upsilon \right)\right]}^{\frac{q}{2}}\right\}}^{\frac{1}{q}},$$

In the fluctuation function *F_q_*(*m*), the moment order *q* can assume any real value other than zero, while for *q* = 0, the fluctuation function is calculated by a logarithmic averaging procedure:

(6) $$F_{0}(m)\equiv \exp\left\{\frac{1}{4N_{m}}\sum_{\nu =1}^{2N_{m}}\ln[F^{2}(m,\nu )]\right\}\approx m^{h_{0}},$$

The large fluctuations of signal are enhanced by the positive values of *q*, while the small fluctuations by the negative values. *F_q_*(*m*) increases with *m* as a power-law if the series is characterized by long-range correlations:

(7) $$F_{q}(m)\approx m^{h_{q}}.$$

Plotting *F_q_*(*m*) on log-log scales and fitting it by a least square method versus *m*, the slope of the fitting line is the so-called generalized Hurst exponent *h_q_*, which represents the scaling properties of the series at a certain moment order *q*. In particular, for stationary series, *h*_2_ is the Hurst exponent *H*. If the series is monofractal, the exponent *h_q_* are nearly constant with *q*, which suggests that the scaling behavior of the small and large variations is approximately identical; however, if the series is multifractal, the exponent *h_q_* decreases with *q*, which suggests that the small and large fluctuations scale differently and the series has a more complex structure.

#### 2.3.2. Fisher–Shannon Analysis

The Fisher–Shannon (FS) method permits the analysis of complex time series by jointly using the Fisher Information Measure (FIM) and the Shannon entropy (SE). The FIM and SE describe the characteristics of the probability density function of a series, respectively, at a local and global scale [24], and are commonly used to investigate the complexity of non-stationary time series in terms of order and organization (FIM) and disorder and uncertainty (SE) [41]. The FIM and SE are calculated, as follows:

(8) $$\mathrm{FIM}=\int_{-\infty }^{+\infty }{\left(\frac{\partial }{\partial x}f(x)\right)}^{2}\frac{dx}{f(x)},$$

(9) $$\mathrm{SE}=-\int_{-\infty }^{+\infty }f_{X}(x)\log f_{X}\left(x\right)dx,$$

where *f*(*x*) is the probability density function of the series *x*. Because SE can also be negative, the exponential transformation of Shannon entropy is generally applied to obtain the so-called Shannon entropy power *N_X_* that is commonly utilized in statistical analysis:

(10) $$N_{X}=\frac{1}{2\pi e}e^{2H_{X}},$$

According to the isoperimetric inequality $\mathrm{FIM}\cdot N_{X}\geq D$ [42], where *D* is the dimension of the space (1 in case of time series), the FIM and the *N_X_* are interrelated, which suggests that a better description of the dynamics of a time series would be given by using jointly both the measures. The equality stands in case of Gaussian processes. The use of both measures provides a more complete description of the time series, since the FIM focuses on the local properties of the probability density function, while the Shannon entropy on the global properties. It was also shown in Martin et al. (1999) [43] that FIM allowed for the detection of some non-stationary behavior in situations where the Shannon entropy showed a limited dynamics. 

As the calculation of FIM and *N_X_* depends on the probability density function, attention has to be paid to its good estimation. In this study, we used the kernel-based approach to estimate *f*(*x*), which has been shown to have a better performance than the discrete-based approach in calculating the value of FIM and SE for the Gaussian distributed series [44]. The kernel-based approach for estimating the probability density function is based on the kernel density estimator technique [45,46]:

(11) $${\hat{f}}_{M}(x)=\frac{1}{Mb}\sum_{i=1}^{M}K\left(\frac{x-x_{i}}{b}\right),$$

where *b* refers to the bandwidth, *M* represents the number of data, and *K*(*u*) is a continuous non-negative and symmetric kernel function that satisfies the following two conditions:

(12) $$K\left(u\right)\geq 0\;\mathrm{and}\;\int_{-\infty }^{+\infty }K(u)du=1,$$

The estimation of *f*(*x*) uses an optimized integrated method [43] that is based on Troudi et al.’s [47] and Raykar and Duraiswami’s [48] algorithms, where a Gaussian kernel with zero mean and unit variance is adopted:

(13) $${\hat{f}}_{M}(x)=\frac{1}{M\sqrt{2\pi b^{2}}}\sum_{i=1}^{M}e^{-\frac{{\left(x-x_{i}\right)}^{2}}{2b^{2}}},$$

The isoperimetric inequality enables the application of the Fisher–Shannon (FS) information plane to explore the dynamics of a series [49], in which the coordinate axes are *N_X_* and FIM. For scalar signals, the line FIM·*N_X_* = 1 divides the FS information plane into two parts, and each signal is represented by a point that lies exclusively in the half-space of FIM·*N_X_* > 1. 

## 3. Results

**Figure 2:**
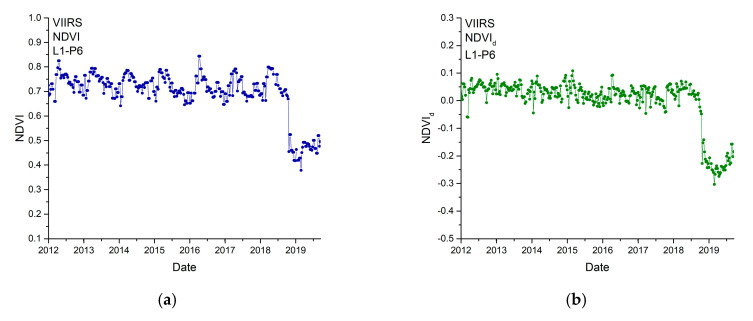

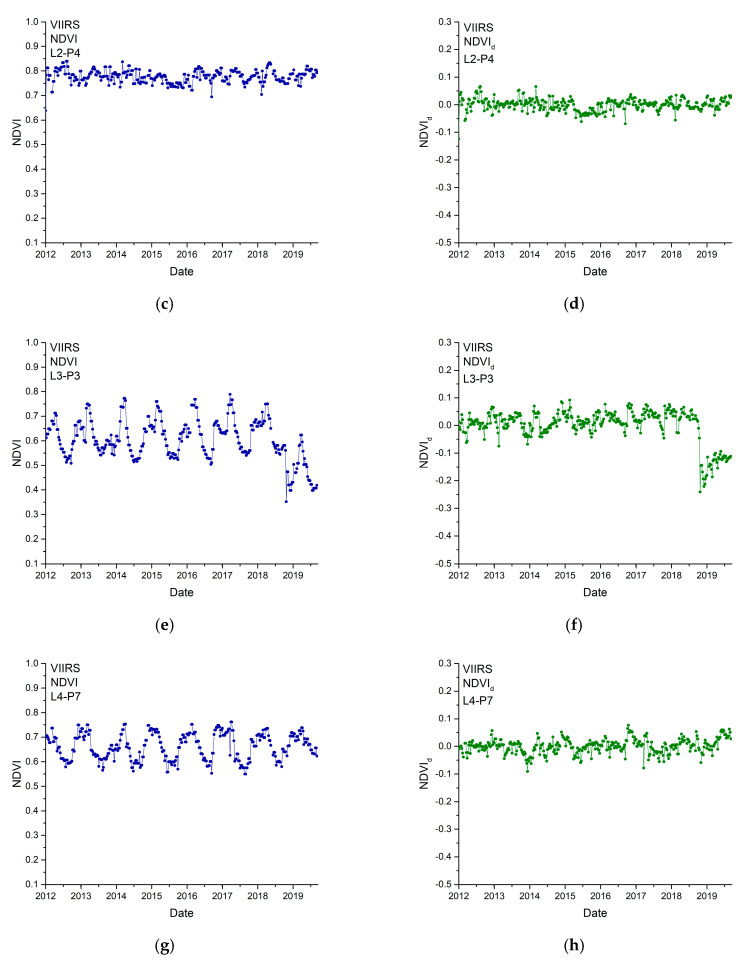
Time series of NDVI and NDVI_d_ of an example pixel for each study site. The NDVI of L1-P6, L2-P4, L3-P3 and L4-P7 are shown in (**a**), (**c**), (**e**), and (**g**), while the NDVI_d_ of which are shown in (**b**), (**d**), (**f**), and (**h**).

We analyzed four study sites, among which two were affected by the Camp Fire (L1 and L3), and two were not affected (L2 and L4), as they were located outside the area burned by the wildfire. According to the data pre-processing procedure of outlier filtering described in Section 2.1, very few outliers were omitted for each site, in particular 2.54% (L1), 2.54% (L2), 1.98% (L3), and 1.98% (L4). L1 and L2 were covered by evergreen needleleaf forests, while L3 and L4 by woody savannas. For each study site we extracted the NDVI time series of 3×3 adjacent pixels (P1 to P9). For each pixel, we calculated NDVI_d_ that we analyzed by the MFDFA and the FS method. Figure 2 shows, as an example, the NDVI and the NDVI_d_ of some pixels of each site. Although the NDVI of needleleaf forests (Figure 2a,c) appears to be higher than that of woody savannas (Figure 2e,g), however the departure NDVI_d_ of both vegetation covers do not show significant difference, thus confirming the effectiveness of the seasonal removal by the procedure of Equation (2). Furthermore, even by visual inspection, it is clearly seen that both NDVI and NDVI_d_ drop sharply down at the time of Camp Fire occurrence, towards increase, then, gradually through time to the pre-fire conditions. 

**Figure 3:**
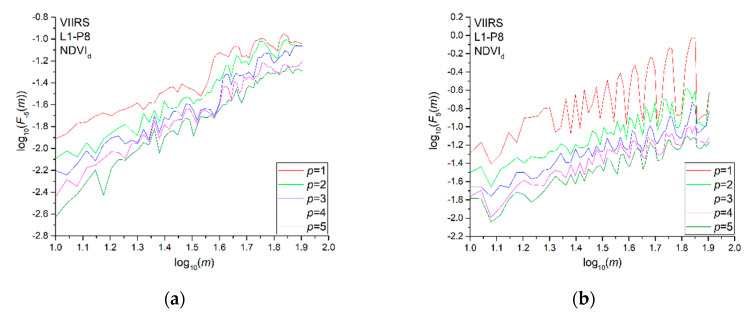
*q*-th order fluctuation function with the polynomial degree *p* ranging from 1 to 5 of NDVI_d_ for the pixel P8 of site L1. (**a**) *q* = −5 and (**b**) *q* = 5.

In order to select the optimal value of *p*, we calculated and compared the fluctuation functions obtained by using different degrees *p* since the MFDFA depends on the degree of the detrending polynomial *p*. Since the detrending of the time series is performed by subtracting the polynomial from the profile, different orders of DFA differ in their possibility to eliminate trends in the series. In MFDFA of order *p*, trends of order *p* are eliminated from the profile. Thus, comparing the fluctuation functions for different orders *p* allows for one to estimate the type of the polynomial trend in the time series and to select the optimal one, which is the order that is enough to remove those trends in the series. Figure 3 shows, as an example, the fluctuation functions for two values of the moment order *q*, −5 (Figure 3a) and 5 (Figure 3b), for the pixel P8 of site L1, for different orders of *p* from 1 to 5. Since the fluctuation functions tend to overlap for value of *p* higher than 4 in both cases, the optimal degree of the detrending polynomial is 4; thus, the multifractal analysis was performed by using a 4th degree detrending polynomial. 

**Figure 4:**
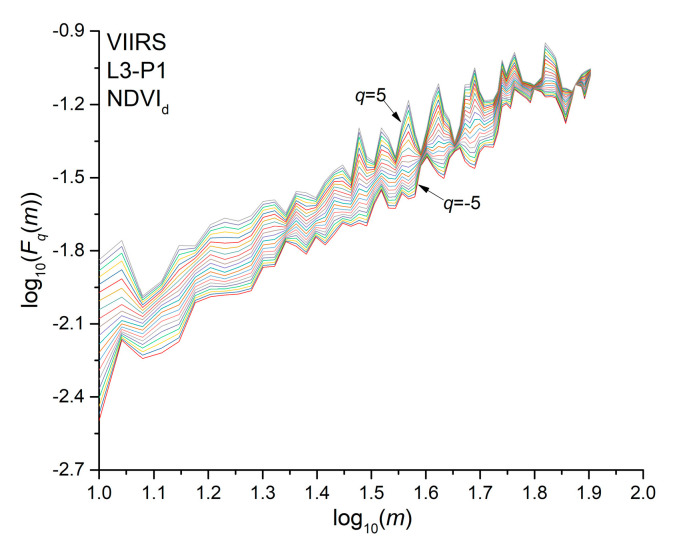
Fluctuation functions with *q* ranging from −5 to 5 of NDVI_d_ for the pixel P1 of site L3.

The multifractality of a time series is revealed by the power-law behavior of the fluctuation functions for any moment order *q*. Plotting the fluctuation function in log-log scales, the multifractality of the series is indicated by the linear behavior of the fluctuation functions, whose slope, estimated by using a least square method, is the so-called generalized Hurst exponent *h_q_*. Figure 4 shows as an example the fluctuation functions of the NDVI_d_ of pixel P1 of site L3 for *q* ranging from −5 to 5.

**Figure 5:**
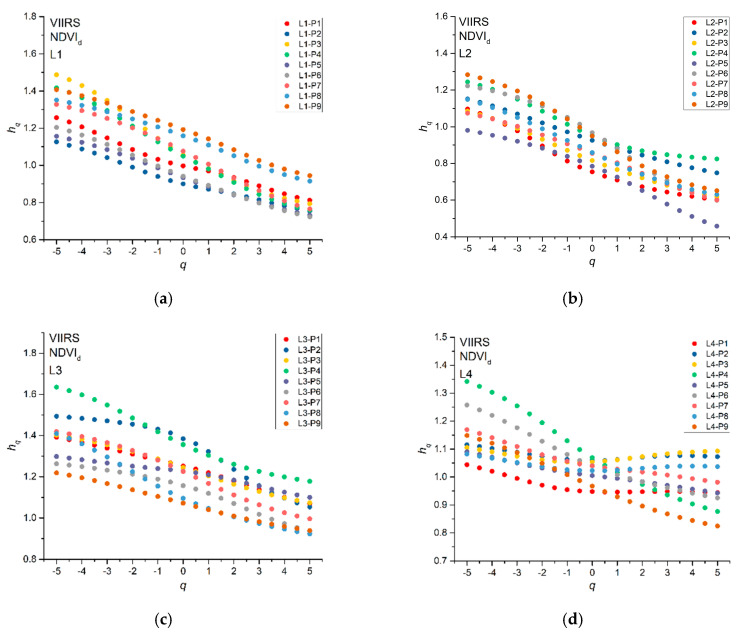
Generalized Hurst exponents *h_q_* of NDVI_d_ for all the pixels of the four study sites L1 (**a**), L2 (**b**), L3 (**c**), and L4 (**d**).

**Figure 6:**
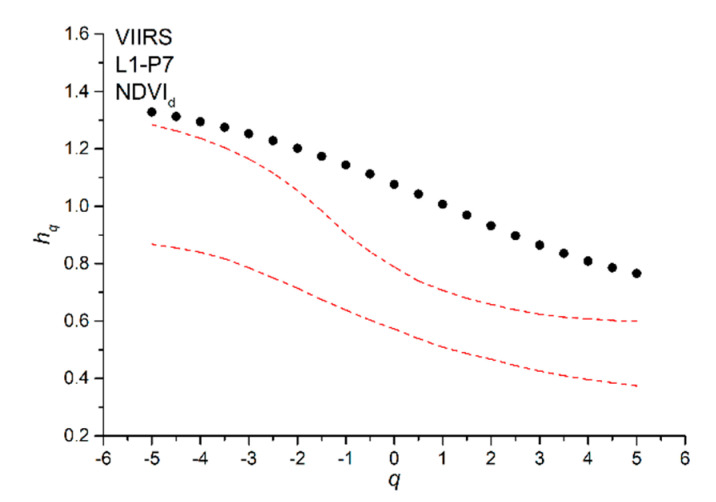
Generalized Hurst exponents *h_q_* of NDVI_d_ for the pixel P7 of site L1 (black dots) and the 95% confidence band of the *h_q_* of the shuffles (red dotted lines).

Figure 5 shows the generalized Hurst exponents *h_q_* that were calculated for all NDVI_d_ series. Most of the *h_q_* decrease with the increase of the moment order *q* for all of the pixels; however, for a few pixels (P1, P2, P3, and P8 of site L4), *h_q_* fluctuates around the average value. We applied the MFDFA to one hundred shuffles for each NDVI_d_ series and calculated their *h_q_* in order to recognize the source of multifractality (long-range correlations or type of distribution). Since the shuffling just removes the correlations but preserves the distribution of the series, we can discriminate between the multifractality due to the existence of long-range correlations (the *h_q_* of the shuffles fluctuates around the value of 0.5) or to the probability density function of the series (the *h_q_* of the shuffles behave in nearly the same manner as those of the original series). Figure 6 shows, as an example, the generalized Hurst exponents *h_q_* (black dots) of NDVI_d_ series and the 95% confidence band (red dotted lines) of the *h_q_* of the shuffles for the pixel P7 of site L1; we can see that the *h_q_* of the original series are well beyond the 95% confidence band of the shuffles, whose generalized Hurst exponents, however, slightly decrease with the moment order *q*; this indicates that the multifractality of the NDVI_d_ series could be due to both the presence of long-range correlations in the series and the distribution of the values.

**Figure 7:**
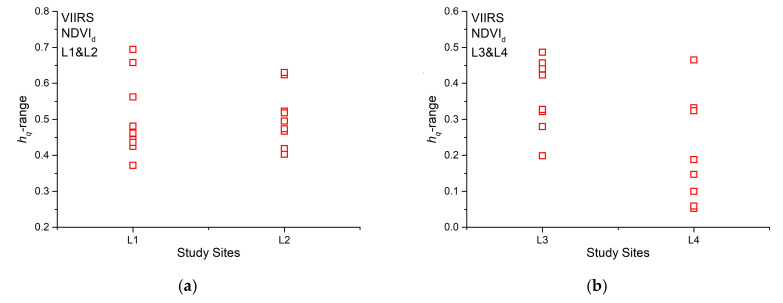
Range of the generalized Hurst exponent (*h*_q_-range) of NDVI_d_ for (**a**) sites L1, L2 and (**b**) sites L3, L4.

We use the range of the generalized Hurst exponent (*h_q_*-range) that is the difference between the maximum and the minimum generalized Hurst exponent to quantify the multifractality. Figure 7 shows the *h_q_*-range of all the analyzed NDVI_d_ series. From a visual inspection, for evergreen needleleaf forests, the hq-range of burned and unburned sites is approximately the same (Figure 7a), while a certain difference seems to appear in the case of savannas (Figure 7b), indicating that the wildfire could have changed the multifractal characteristics of savannah, but not for evergreen needleleaf vegetation. In order to check whether the averages of *h_q_*, <*h_q_*>, are significantly different, we applied the two sample Student’s t-test to the group of *h_q_* calculated for L1 and L2 and for L3 and L4. We found that at 95% confidence, L1 and L2 are not significantly distinguishable (*p*-value = 0.381106), while L3 and L4 are significantly distinguishable (*p*-value = 0.010356), which indicates that the fire-affected and fire-unaffected sites are distinguishable for savanna vegetation covers, and not for evergreen needleleaf forests.

**Figure 8:**
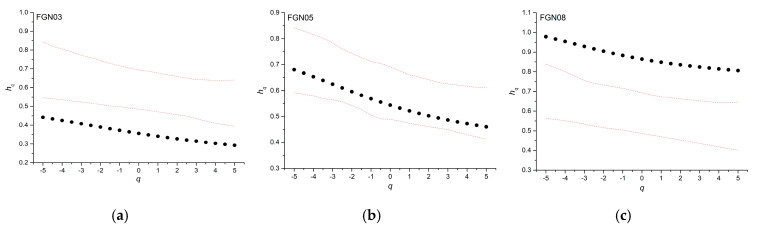
Generalized Hurst exponent of the Fractional Gaussian Noise simulations with three different Hurst exponent values of (**a**) 0.3, (**b**) 0.5, and (**c**) 0.8.

We calculated the generalized Hurst exponents for three simulations of Fractional Gaussian Noise (FGN) with three different values of the Hurst exponent (0.3, 0.5, and 0.8), indicated respectively as FGN03, FGN05, and FGN08, and same length as our time series, in order to check the robustness of the results against the short length of the time series (395 values). Figure 8 shows the generalized Hurst exponents and their 95% confidence band based on one hundred shuffles. Although the FGN is a monofractal process, its simulations show a slight multifractality that is probably due to the short length. However, their *h_q_*-range is 0.15 for FGN03, 0.18 for FGN05, and 0.16 for FGN08; the small value of the *h_q_*-range confirms the monofractal character of the three time series. Furthermore, the generalized Hurst exponents of FGN03 and FGN08 are well beyond the 95% confidence band that is based on the shuffles, and this strengthens the robustness of the MFDFA in detecting multifractality, even in short time series.

Although the pixels extracted for each site are spatially located close to each other, a certain local variability could still exists that might influence the multifractal character of the NDVI_d_; thus, we also analyzed the mean NDVI_d_, <NDVI_d_>, which is the mean of NDVI_d_ over the nine pixels for each site, and that might represent the behavior of vegetation in a site in a more global manner.

**Figure 9:**
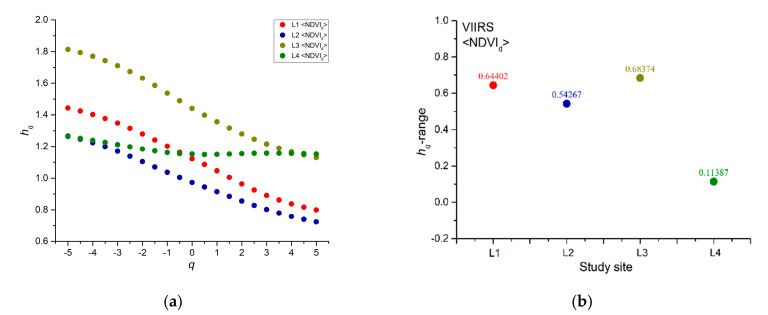
Generalized Hurst exponents *h_q_* (**a**) and *h_q_*-range (**b**) of the <NDVI_d_>.

Figure 9 shows the generalized Hurst exponents *h_q_* and the *h_q_*-range of the <NDVI_d_> of the four sites. A general pattern seems to be evidenced from the analysis of <NDVI_d_>; in fact, for both the evergreen needleleaf forests and woody savannas, the <NDVI_d_> is characterized by a value of *h_q_*-range for burned sites (L1 and L3) larger than that of unburned sites (L2 and L4), although for savanna covers the difference between the burned and unburned sites is much larger.

**Figure 10:**
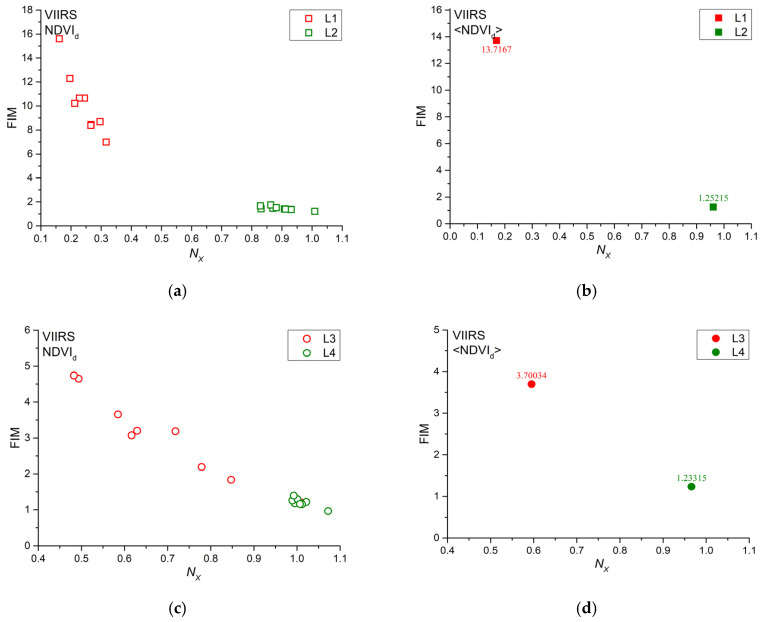
Fisher–Shannon information plane for the (**a**) NDVI_d_ and (**b**) <NDVI_d_> comparisons of L1 and L2, (**c**) NDVI_d_, and (**d**) <NDVI_d_> comparisons of L3 and L4.

The Fisher–Shannon method was applied to all of the investigated NDVI_d_ series and the results are shown in the Fisher–Shannon information plane (Figure 10). It can be seen that a clear separation exists between the burned and unburned sites for both vegetation covers. The two sample Student’s t-test for the *N_X_* and FIM indicates a significant difference between the two groups of the obtained parameters of burned and unburned sites (*p*-value < 0.05) (Table 1).

## 4. Discussion

The NDVI is one of most widely used vegetation indices for characterizing the vegetation status. The NDVI can also be used to discriminate between different types of vegetation covers; in our case, the variability of NDVI is smaller for evergreen needleleaf forests and larger for woody savannas. The NDVI is very sensitive to situations where the vegetation is stressed, like when being affected by a wildfire. In this study, we analyzed the vegetation dynamics of two different vegetation covers to investigate the impact of fire. We used two different methods, the MFDFA and the FS method, obtaining a set of parameters (*hq*-range, FIM and *N_X_*), by which the discrimination between burned and unburned sites was investigated.

The *h_q_*-range is used to quantify the degree of multifractality of a series. A larger multifractality indicates that the series is characterized by a larger heterogeneity that means the large fluctuations of the series scale differently from the small fluctuations since the MFDFA highlights the scaling behavior of the time dynamics of a series. The NDVI of unburned sites of savannas type are featured by a relatively small value of *h_q_*-range, which indicated that the NDVI is rather monofractal and the scaling behavior of the large and small variations is nearly the same; the vegetation is probably only subjected to fluctuations induced by the seasonal and climatic effects. The NDVI of fire-affected sites of woody savannas covers appear, instead, more heterogeneous, suggesting that small and large fluctuations scale differently; the wildfire impacted on this vegetation that responded to such stress by intermittently fluctuating and thus increasing its multifractality. Such a difference between burned and unburned sites covered by woody savannas becomes much more evident when analyzing the <NDVI_d_>, which might more globally represent the status of vegetation of a site, filtering out the local variability that could affect single pixels. The <NDVI_d_> of woody savannas, in fact, allows for very clearly discriminating between burned and unburned sites, with the last being signaled by a lower multifractality degree. Additionally, the <NDVI_d_> of unburned sites of evergreen needleleaf forests is characterized by a relatively lower multifractalty degree than burned sites, although no significant difference has been found between the single pixels of fire-affected and fire-unaffected sites. Such not so clear multifractal difference between burned and unburned sites that are covered by evergreen needleleaf forests could be due to the fact that fire mainly affects the grass, whose vegetation fine texture makes it more sensitive to the burning than is true of leaves and stems of forests, as was observed in [50].

The FS information plane allows for investigating the complex structure of a time series in terms of organization/order. Generally speaking, the bottom right part of the FS plane is occupied by series characterized by less ordered and less organized series, while the top left part of it by more ordered and more organized series. In the FS information plane, the pixels of the burned sites are well discriminated from those of the unburned sites for both evergreen forests and woody savannas. In particular, pixels of burned sites have smaller *N_X_* and larger FIM values, whereas those of the unburned sites are characterized by larger *N_X_* and smaller FIM in agreement with previous researches [21]. A larger FIM and a smaller *N_X_* quantifies a larger organization and less disorder of the series, where, for larger organization and less disorder, we mean that the distribution of the values of the series is rather peaked, and some values are more frequent than others. Since the vegetation undergoes the disturbance of wildfire, the sudden decrease of NDVI soon after the wildfire and its gradual increase during the post-fire vegetation recovery makes the distribution of the values less uniform than that characterizing the NDVI of the unburned sites; thus, the vegetation does not uniformly fluctuate and the FIM and *N_X_* are larger and smaller, respectively.

## 5. Conclusions

The Suomi-NPP VIIRS NDVI series of two types of vegetation covers were analyzed using the multifractal detrended fluctuation analysis and the Fisher–Shannon method. Pixel time series of burned and unburned sites were investigated. Several parameters, in particular the range of the generalized Hurst exponent (*h_q_*-range), Shannon entropy power (*N_X_*), and Fisher Information Measure (FIM), were utilized to measure the degree of heterogeneity and organization of the vegetation series in conjunction with their separating ability to discriminate fire-affected and fire-unaffected pixels.

In general, the NDVI of the fire-affected sites are more heterogeneous in comparison to that of the fire-unaffected sites, especially for woody savannas. In addition, the vegetation dynamics of fire-affected sites are characterized by a larger organization and smaller disorder, due to the less uniform behavior that characterizes vegetation when it is stressed by a fire and during its post-fire recovery process.

Furthermore, the FS information plane has revealed its potential in being sensitive to wildfire impact on different types of vegetation more than MFDFA; in fact, the MFDFA has allowed for discriminating the vegetation dynamics between the burned and unburned sites more significantly in woody savannas covers than in evergreen needleleaf forests.

## Figures and Tables

**Table 1:** *p*-value of Student’s t-test for the *N_X_* and FIM of NDVI_d_.

	L1–L2	L3–L4
*N_X_*	1.4743E−14	0.000431
FIM	0.000007	0.001475

## Abbreviations and Nomenclatures

NDVI	Normalized Difference Vegetation Index
MFDFA	multifractal detrended fluctuation analysis
FS	Fisher-Shannon
VIIRS	Visible Infrared Imaging Radiometer Suite
Suomi-NPP	Suomi National Polar-Orbiting Partnership
MODIS	Moderate Resolution Imaging Spectroradiometer
EOS	Earth Observing System
VI	vegetation indices
ROI	region of interest
DFA	Detrended Fluctuation Analysis
FIM	Fisher Information Measure
SE	Shannon entropy
*N_X_*	Shannon entropy power
BRDF	bidirectional reflectance distribution function
SDS	science data sets
NDVI_d_	departure NDVI
PG&E	Pacific Gas and Electric Co.
IGBP	International Geosphere-Biosphere Programme
*h_q_*	Generalized Hurst exponents
*h_q_*-range	range of the generalized Hurst exponent
FGN	Fractional Gaussian Noise
